# The Effect of a Mineralized Bone Graft on the Surface Microhardness of Mineral Trioxide Aggregate and Biodentine 

**DOI:** 10.22037/iej.v13i1.14953

**Published:** 2018

**Authors:** Saeed Rahimi, Shahriar Shahi, Zahra Torabi, Yashar Rezaie, Negin Ghasemi, Somayeh Abolhasani

**Affiliations:** a *Department of Endodontics, Dental and Periodontal Research Center, Dental School, Tabriz University of Medical Sciences, Tabriz, Iran; *; b *Dental School, Tabriz University of Medical Sciences, Tabriz, Iran; *; c *Department of Biostatistics, Dental School, Tabriz University of Medical Sciences, Tabriz, Iran*

**Keywords:** Biodentine, Bone Graft Materials, Mineral Trioxide Aggregate, Vickers Microhardness Test

## Abstract

**Introduction:**

This study was designed to determine the effect of Osteon II mineralized bone powder on the surface microhardness of two retrofilling materials: Mineral trioxide aggregate (MTA) and Biodentine (BD).

**Methods and Materials:**

Each retrograde material was mixed and carried into 30 sterile custom-made plastic cylinders. Half of the samples in each group were exposed to Osteon II. All cylinders were submerged in simulated tissue fluid and incubated at 37^°^C and 100% relative humidity for 7 days. Surface microhardness values of each study group was attained using Vicker’s microhardness test. The data were analyzed statistically using two-way ANOVA and independent* t* test at a significance level of 0.05.

**Results:**

In all the setting conditions, BD had significantly greater surface microhardness than MTA (*P*<0.001). Surface microhardness of both materials was significantly reduced in the presence of osteon II (*P*=0.006 for BD and *P*<0.001 for MTA).

**Conclusion:**

Mineralized bone graft materials negatively affect surface microhardness of both MTA and BD. In presence of osteon II, BD had the highest surface microhardness.

## Introduction

Endodontic surgery is necessary when conservative root canal treatment fails to provide complete apical seal or is not feasible. It is also indicated as the last treatment option for non-healing large periapical lesions before extraction [1]. In such procedures, retrograde materials are used which should ideally prevent microleakage to seal the apex, should be bioactive and biocompatible, should exhibit dimensional stability and antibacterial properties and should at the same time have proper mechanical properties at close proximity to the periapical tissues [2].

Of all the materials which have been introduced as retrofilling materials, none has all the above-mentioned properties and studies are underway to find a superb retrofilling material to achieve the best clinical results. In this context, mineral trioxide aggregate (MTA) which is silicate cement with many applications in the field of endodontics has gained popularity. However, difficult handling and long setting time are known disadvantages of MTA [3]. Another concern in relation to this material is vulnerability of its hydration process to the materials found at the endodontic surgical site [4]. The extent and quality of the setting of material can be studied with the use of different techniques, including surface microhardness or the ions released from the material under study [5, 6].

Another environmental factor that should be taken into account in the real environment of periapical surgery is the presence of bone powders. In endodontic surgeries that are associated with large osseous lesions use of bone powders is common clinically to accelerate tissue healing and strong evidence has been reported on the use of such materials [7]; however, it has been shown that the surface microhardness of MTA, which is the most commonly used retrofilling material, decreases significantly in the presence of mineralized bone powders. The mechanism of this effect has not been elucidated definitely; however, it is possible that ions present in the bone powder are involved in such an effect [8]. A decrease in microhardness can result in an increase in solubility and decrease in the apical seal [9].

Biodentine (BD) is a relatively new calcium silicate cement with a high rate of purity and has been claimed to replace dentin in relation to its mechanical properties. This material is marketed in single-dose capsules which mainly consist of tricalcium silicate and calcium carbonate and a small amount of zirconium oxide to provide radiopacity. Its powder and liquid are mixed in an amalgamator for 30 sec and then used. Its setting time has been reported to be 12 min [10].

BD is used in the clinic for several purposes, including root perforations, apexification, pulp capping procedures and as a retrofilling material [[11]]. Compared to MTA, BD exhibits better workability and histopathological studies have shown its favorable level of biocompatibility [[12, 13]]. In addition, this material exhibits bacteriostatic properties [[14]]. The effect of an acidic environment on the surface microhardness of BD has been compared with MTA, with BD yielding better results. Therefore, BD is recommended under acidic conditions [[15]].

No data is available on the effect of bone powders on the surface microhardness of BD and no comparison has been made to date between changes in surface microhardness of BD and MTA in the presence of bone powders. Therefore, the aim of the present study was to determine the effect of Osteon II bone powder on the surface microhardness of MTA and BD. The present study was undertaken based on the null hypothesis that Osteon II bone powder (Genoss, Suwon, Korea) has no effect on the surface microhardness of these two retrofilling materials.

## Materials and Methods

A total of 60 custom-made sterile plastic cylinders with an internal diameter of 3 mm and a height of 2 mm were prepared. The materials investigated in this study were: mineral trioxide aggregate (MTA; Angelus, Londrina, Paraná, Brazil) and Biodentine (BD; Septodont, St. Maur-des-Fossés, France). Both materials were mixed and prepared based on manufacturers’ instructions. A powder-to-liquid ratio of 3:1 was used for MTA. The powder and liquid of BD were mixed according to manufacturer’s instructions in an amalgamator for 30 sec.

Each material was carried into 30 cylinders and then condensed against a glass surface by a single operator. An amalgam knife was used to produce an even and smooth surface for the material with plastic cylinders and remove excess material. Physical damage of condensed surface was avoided. The filled cylinders were randomly assigned to control and experimental groups, placed in a 64-well plate and categorized into 4 experimental groups with different environments: Group 1, 15 cylinders of MTA in simulated tissue fluid (STF); Group 2, 15 cylinders of BD in STF; Group 3, 15 cylinders of MTA in STF and exposed to Osteon II and Group 4, 15 cylinders of BD in STF and exposed to Osteon II.

Each cylinder was submerged in STF. STF was prepared according to the method suggested by Shahi *et al.* [16]. Group 3 and group 4 samples were indirectly exposed to 0.1 mg of synthetic biphasic calcium phosphate graft material (Osteon II, Genoss, Suwon, Korea). The plate was sealed to prevent dehydration and vaporization and was incubated for 1 week at body temperature. After incubation, the cylinders in each group were mounted on acrylic plates measuring 3 mm in height with the condensed sides facing out. The mounted samples were wet-polished using silicon carbide sandpapers from 400 to 2000 grit, respectively. Finally, the polished acrylic plates were washed with distilled water and dried at room temperature.

Surface microhardness of all the groups was determined using Vickers microhardness test performed by UHL-VMHT microhardness tester (WalterUhl, Asslar, Germany) with a load of 300 g and a dwell time of 10 sec. Three indentations were created on each cylinder with a distance of at least 1 mm on the polished and microscopically sound surface of the material. Indentations were placed at least 1 mm far from cylinder periphery. The machine digitally calculated microhardness values after each indentation based on Vickers microhardness formula. (VHN=2Fsin (136^º^/2)/d^2^= 1.854F/d^2^)

***Statistical analysis***

The mean value of the three indentations on each cylinder was obtained. Data was collected and analyzed with SPSS software (Statistical Package for Social Science, SPSS, version 16.0, SPSS, Chicago, IL, USA).

Kolmogorov-Smirnov test was used to confirm normal distribution of the data and normality was verified (>0.05). Mean and standard deviation of surface microhardness values of each group were obtained and compared statistically by means of two-way ANOVA after homogeneity of variances was confirmed with Levene’s test (*P*>0.05). Independent *t* test was used within each study group at a significance level of 0.05.

## Results

Table 1 presents the results of surface microhardness test for each study group. Descriptive analyses of data showed the highest microhardness for BD in the group in which Osteon II was absent, with the lowest related to MTA in the presence of Osteon II. 

It was shown that both independent variables of the type of retrofilling material and the presence of bone powder in the environment caused significant changes in surface microhardness at different levels (*P*<0.001). The interaction effect of these variables was not significant (*P*=0.08). Effect of presence or absence of Osteon was reported 22% whereas the effect of type of retrofilling material was reported 86% and observed power was 1%. Adjusted R squared value was 0.86 which means that almost 86% of changes in microhardness value could be predicted by these two variables (Table 2).

Considering the normal distribution of data, to compare the mean microhardness values separately for the two material types in the presence and absence of bone powder, *t *test was used, which showed significant differences in microhardness values (*P*=0.006 for BD and *P*<0.001 for MTA). Comparison of the means between the study groups showed that BD was affected more profoundly by the presence of bone powder, with decrease of 18.4 units in surface microhardness compared to a decrease of 7.2 units in surface microhardness of MTA

## Discussion

The present study was undertaken to compare the surface microhardness of MTA and BD, two retrofilling materials, in the presence and absence of mineralized bone powder. The results showed that the surface microhardness of BD was significantly higher than that of MTA. Based on the results of the present study, the presence of synthetic Osteon II bone powder, which is one of the bone powders in the biphasic calcium phosphate bone graft materials, can exert a negative effect on the surface microhardness of both retrofilling materials, which is significant statistically. The effect on BD was more than that on MTA in the presence of bone powder; however, BD still exhibited surface microhardness 3 times that of MTA in the presence of Osteon II.

MTA has high biocompatibility and has repeatedly been used as a material with known properties in endodontic surgeries [3]. Therefore, in the present study, this material was used.

Based on evidence, the surface microhardness of materials shows their degree of setting in different environments and it reflects the strength of the materials in general [5]. Vickers’ hardness test is widely used to determine such physical property. Considering the frequent use of this test in previous related articles [15, 17], it is possible to compare the results of these studies with the current study.

In this *in vitro* study, efforts were made to simulate the environmental conditions of periapical surgery as much as possible; the thickness of the samples was 3 mm because previous studies have shown that the thickness of the retrofilling material can affect its hardness [18]. Considering the clinical recommendations in relation to the minimum of 3 mm of thickness for retrofilling materials in retrograde surgeries to control microleakage, this thickness was used in the present study [19].

In contrast to similar studies on the subject [8], plastic was chosen over methyl methacrylate for cylinders to avoid possible monomer release which might affect the physiochemical properties of study materials. 

Studies have shown that differences in condensation forces can affect the surface microhardness of retrofilling materials [20]. In order to prevent bias, all the samples in the present study were prepared by one operator with the use of same instrument for condensation. The samples were incubated at 37^°^C and 100% relative humidity for 1 week and during this period the samples were shaken gently to create a homogeneous ionic environment in test plates. All the samples in the study groups were immersed in an environment containing STF, which has been reported to have similar ionic composition to interstitial and dentinal fluid [21]. 

**Table 1:** Descriptive statistics for Vickers surface microhardness values of the study groups

**Retrofilling material (N)**	**Osteon Presence**	**Mean (SD)**	***P*** **-value**
**Biodentine**	yes	80.5747 (13.49)	0.447
	no	99.0480 (19.85)	
**MTA**	yes	26.0433 (4.23)	0.278
	no	33.3094 (4.38)	

**Table 2:** Tests of Between-Subjects Effects-two way ANOVA (Adjusted R Squared=0 .86**)**

**Source**	**F Statistic**	**Partial Eta Squared**	**Observed Power**	***P*** **-value**
**Corrected Model**	127.728	0.871	1	<001
**Intercept**	1440.157	0.962	1	<001
**Retrofil**	364.769	0.865	1	<001
**Osteonpresence**	16.707	0.227	0.98	<001
Retrofil * Osteonpresence	3.167	0.053	0.417	0.08

Moreover, ions in the tissue fluids can affect the complex hydration processes and crystallization of the retrofilling material during setting [3]. In accordance with Sato *et al.* [3]. In accordance with Sato *et al.* [8] we used immersion technique instead of using sponges soaked in STF to provide improved wetting of the retrofilling material. STF solution has a great role in simulating the periapical surgical area by providing the moisture of the setting environment and through its content of tissue fluid ions.

The higher surface microhardness of BD compared to MTA in the present study is consistent with the results of previous studies [17, 22]. It has been suggested that higher hardness of BD is related to its low liquid-to-powder ratio [22, 23]. The manufacturer has incorporated water-soluble polycarboxylate polymer into it to provide high workability despite its low liquid-to-powder ratio [24]. The claims of the manufacturer in relation to the potential of BD to restore the strength of sound dentin can be confirmed by comparing the results of previous studies on the surface microhardness of sound dentin, which has been reported at a range of 60-90 VHN, and the measurements carried out in the simulated environment of the present study [17, 25, 26]. No studies to date have evaluated the effect of bone powders on the setting of BD. In the present study Osteon II bone powder, which is considered a mineralized bone powder, was used. Sato *et al.* [8] reported that mineralized and demineralized bone powders can affect the surface microhardness of WMTA differently. They showed that the surface microhardness of this retrofilling material after one month in the presence of demineralized bone powder was not different from that in the control group; however, the presence of mineralized bone powder, possibly due to the release of ions into the setting environment of the retrofilling materials and their interference with the hydration process, decreased surface microhardness significantly [8]. In the present study, too, such an effect on MTA and BD was observed and confirmed. Based on the results of the present study, despite the detrimental effects of bone powders on the surface microhardness of retrofilling materials, BD was a better material compared to MTA due to a three-fold higher surface microhardness.

It should be pointed out that the effect of blood contamination and the periapical tissue inflammatory conditions on retrofilling materials in the present study was not simulated and evaluated. The presence of inflammation and the resultant severe decrease in pH is inevitable in the endodontic surgery environment. Both BD and MTA exhibited lower hardness at low pH values, which has been explained in different studies by interference with the setting process, a decrease in adhesion and an increase in the solubility of these cements in the acidic environment. It was suggested in a study by Elnaghy *et al.* [15] that BD be used instead of White MTA in the presence of inflammation.

Contamination with blood or serum is another factor that can affect retrofilling materials surface microhardness under clinical surgical conditions. Despite the use of different hemostatic techniques, blood contamination of the endodontic surgical environment, especially in the vicinity of large periapical lesions, is inevitable. To evaluate such an effect both serum and blood have been used. In general, the results showed a severe decrease in microhardness of MTA in the presence of simulated blood contamination [27]. Evaluation of the microscopic structure showed that blood interferes with the formation of acicular crystals [28].

A number of researchers have used the force of ultrasonic devices in order to apply uniform condensation pressure and have concluded that use of this technique can result in a higher surface microhardness [29]. Ignoring such a parameter can be considered one of the limitations of the present study.

The follow-up period in the present study was one week, which can be considered a limitation of this study. Further studies are necessary in relation to the time required to follow the results. In a study by Butt *et al.*, [30] there were no significant changes in the mechanical properties of BD between the 1-week and 3-month intervals; however, it was not evaluated whether or not there were changes in the surface microhardness of BD after one week in the presence of mineralized bone powders. In relation to MTA, studies have shown that the hardness of this material might increase over time [8, 29].

Making a decision about the material which is superior for retrograde surgeries is not possible only through comparison of surface microhardness. Microhardness was evaluated only as one of the mechanical properties of retrofilling materials and as a criterion for the evaluation of their strength.

## Conclusion

Mineralized bone graft materials negatively affect surface microhardness of both MTA and BD. In presence of osteon II, BD had the highest surface microhardness.
